# Sphingosine 1-phosphate receptor 1 (S1PR1) agonist CYM5442 inhibits expression of intracellular adhesion molecule 1 (ICAM1) in endothelial cells infected with influenza A viruses

**DOI:** 10.1371/journal.pone.0175188

**Published:** 2017-04-11

**Authors:** Hao Jiang, Si-mei Shen, Jie Yin, Peng-peng Zhang, Yi Shi

**Affiliations:** 1 Department of Respiratory and Critical Care Medicine, Jinling Hospital, Nanjing University School of Medicine, Nanjing, China; 2 Department of Emergency Medicine, the Second Affiliated Hospital, Southeast University, Nanjing, China; The University of Chicago, UNITED STATES

## Abstract

**Background:**

Influenza A virus infection and its complications effect a large population worldwide. Endothelial cells are an important component in lung inflammation caused by influenza A virus infection. The roles of endothelial sphingosine 1-phophate receptor 1 (S1PR1) in the regulation of molecules involved in leukocyte recruitment during influenza A virus infection still remain unknown. In this report, we tested our hypothesis that S1PR1 agonist CYM5442 inhibits expression of intracellular adhesion molecules 1 (ICAM1) in endothelial cells infected with influenza A virus.

**Methods:**

Human pulmonary microvascular endothelial cells (HPMEC) were infected with influenza A virus H1N1. Expression of cytokines, chemokines, interferons, and cellular adhesion molecules was measured by q-PCR. Expression of ICAM1 was further tested by Western Blotting. A S1PR1 agonist CYM5442 was added to the culture media to assess CYM5442’s inhibitory effects during virus infection.

**Results:**

HPMEC could be infected with H1N1 and responded to produce pro-inflammatory cytokines, chemokines, type I interferons, and cellular adhesion molecules. Addition of CYM5442 in culture media reduced the production of ICAM1 via a dosage- and time-dependent manner. CYM5442 inhibited the activation of nuclear factor (NF)-κB. The regulatory effects of CYM5442 were β-arrestin2-dependent.

**Conclusion:**

Activated S1PR1 signaling regulates the production of cellular adhesion molecules by inhibiting NF- κB activation via a β-arrestin2-dependent manner.

## 1. Introduction

Influenza A viruses are a group of pathogens with highly contagious features infecting both human and animals. Influenza virus infection causes seasonal epidemics annually, and pandemic occasionally worldwide. The therapeutic strategies for influenza infection remain suboptimal. Approximately, one of ten human beings are affected by influenza viruses, and 250,000 cases of death are caused by influenza virus infection every year [1]. Despite of the intrinsic factors of influenza viruses [2], excessive pro-inflammatory responses of the host (named the cytokine storm) are also responsible for morbidity and mortality caused by influenza virus infection [3]. Therefore, the regulation of immune responses might be a key to improve the prognosis of influenza virus infection.

Endothelial cells are an important component in inflammation. Endothelial cells that are activated by stimuli recruit leukocytes into inflamed sites, control the blood flow, and leak plasma-protein-rich fluid into the tissue [4]. As the initial cell type infected by influenza A viruses in the respiratory system is epithelial cells, and the average distance between lung epithelial cells and capillaries is only 0.5 μm [5], it is likely that endothelial cells can be affected by influenza A virus through a damaged epithelium. During infection induced by influenza A virus, endothelial cells in lungs are a major source of pro-inflammatory cytokines [6]. More importantly, pulmonary endothelial cells control leukocyte recruitment into lungs during influenza A virus infection by up-regulating cellular adhesion molecules, including intracellular adhesion molecule (ICAM)1, vascular cell adhesion molecule (VCAM)1 and E-selection [7, 8]. Newly recruited leukocytes produce more pro-inflammatory cytokines, and damage the infected lung tissue via many mechanisms [9]. Therefore, manipulating the expression of cellular adhesion molecules on the surface of endothelial cells in the context of influenza A virus infection might control the number of leukocytes involved in lungs and, then the severity of cytokine storm.

Sphingosine 1-phosphate (S1P) is a lipid regulator in several physiologic processes including immune responses [10, 11]. S1P binds to the specific G protein-coupled receptors (GPCRs) termed sphingosine 1-phosphate receptors 1 to 5 (S1PR1-5), and subsequently regulates various cellular effects. Among these abovementioned receptors, S1PR1 signaling in endothelium inhibits vascular permeability and leukocytes transmigration to regulate inflammation [12]. It has been reported that a specific S1PR1 agonist CYM5442 reduces mortality induced by influenza A virus infection via inhibiting the productions of pro-inflammatory cytokines and recruiting leukocytes [6]. However, whether this S1PR1 agonist inhibits the expression of cell adhesion molecules in pulmonary endothelial cells upon influenza A virus infection and the molecular mechanisms underneath this inhibition remain unclear. Here, in the current study, for the first time, we report the S1PR1 specific agonist CYM5442 inhibit the expression of ICAM1 in human pulmonary microvascular endothelial cells (HPMEC) infected by influenza A virus as well as pro-inflammatory cytokines and chemokines via suppressing NF-κB activation by upregulating β-arrestin 2. Thus, these observations shed a light on new mechanisms behind S1PR1-mediated attenuation of cytokine storm and leukocyte infiltration during influenza A virus infection.

## 2. Materials and methods

### 2.1 Reagents and antibodies

The S1PR1 specific agonist CYM5442 was purchased from Sigma-Aldrich (MO, USA). Antibodies against ICAM1, total p65 subunit of NF-κB were purchased from Santa Cruz Biotechnology (TX, USA). Anti-p-p65 subunit, β-arrestin 2, and β-actin antibodies were obtained from Cell Signaling (MA, USA).

### 2.1 Cells and cell culture

Primary human pulmonary microvascular endothelial cells (HPMEC) were obtained from Lonza (CA, USA). HPMEC were cultured in EBM-2 medium with recommended supplements from the supplier and used in passages 6–9. The Madin-Darby canine kidney (MDCK) cell line was purchased from American Type Culture Collection (ATCC, VA, USA). MDCK were cultured in Dulbecco’s Modified Eagle’s medium: Nutrient Mixture F12(DMEM/F12, both from Thermo Fisher Scientific, MA, USA), containing 10% fetal bovine serum (FBS, Thermo Fisher Scientific), and 1× Penicillin-Streptomycin (Thermo Fisher Scientific). Human embryonic kidney HEK293 cells (ATCC, VA, USA) were cultured for screening the effectiveness of knock down process. Human lung epithelial cells A549 were purchased from ATCC, and cultured in RPMI 1640 (Thermo Fisher Scientific) with 5% FBS. Cells were incubated in a humidifier incubator at 37°C with 5% CO_2_.

### 2.3 Virus and viral infection

Influenza virus A/Nanjing/108/2009 (H1N1, referred to as H1N1 below) was isolated and cultured in MDCK cells as described before [13]. The experiment proposal was reviewed and approved by the ethics committee of Jinling Hospital, Nanjing University School of Medicine (Approval Number: JLYY: 2013021). Briefly, H1N1 was grown in 9-day-old embryonated chicken eggs. H1N1 allantoic fluid (VAF) was harvested 48 h post inoculation, and then titrated with standard heamagglutination tests (HA) and plaque assays in MDCK cells. HPMEC cells or A549 cells (5×10^6^ per plate) were cultured in 6-cm petri dishes 12 h prior to viral infection. HPMEC cells were infected with H1N1 inocula in VAF at a multiplicity of infection (MOI) of 1. Virus incula were removed after 1 h of co-incubation, and immediately 3 ml of EBM-2 without serum was added. Infected HPMEC cells were cultured at 37°C with 5% CO_2_ for different periods of time. Cells infected with influenza A H1N1 virus were treated with different concentrations of CYM-5442 or vehicle (PBS) for desired time.

### 2.4 Real-time PCR

Cultured HPMEC cells were collected, and the total RNA was isolated by using a commercial RNeasy mini kit (QIAGEN, CA, USA). A total amount of 2 μg of RNA was used for reverse-transcription to get cDNA with an RT-PCR kit (Thermo Fisher Scientific, MA, USA) following the instruction. Real-time analysis for tumor necrosis factor (TNF)-α, interleukin (IL)-1β, IL-6, CCL1, CCL2, CXCL1, CXCL-8, interferon (IFN)-α, IFNβ, intracellular adhesion molecule (ICAM)1, vascular adhesion molecule (VCAM)1, E-selectin, CD31 and housekeeping gene HPRT was performed by SYBR Green Master Mix (Thermo Fisher Scientific, MA, USA) according to the manufacturer’s instructions. Relative mRNA expression values of interested genes were normalized by the housekeeping gene HPRT. The fold change of mRNA expression was calculated by the equation: 2^(ΔCt of gene-ΔCt of HPRT)^. The melting curve for each interesting gene was analyzed to ensure the specificity of each amplification. The sequences of primers were summarized in Table 1.

**Table 1:** Primer sequences for quantitative Real-time PCR.

Target gene	Direction	Sequence
*TNFα*	Forward	5’TCAACCTCCTCTCTGCCATC3’
	Reverse	5’CCAAAGTAGACCTGCCCAGA3’
*IL-1β*	Forward	5’TGAAATGATGGCTTATTACAGTGG3’
	Reverse	5’GTAGTGGTGGTCGGAGATTCGTAG3’
*IL-6*	Forward	5’CACACAGACAGCCACTCACC3’
	Reverse	5’TTTTCTGCCAGTGCCTCTTT3’
*CCL1*	Forward	5’GGACACAGTTGGATGGGTTC3’
	Reverse	5’GTAGTTTCGGGGACAGGTCA3’
*CCL2*	Forward	5’CTTCTGTGCCTGCTGCTCAT3’
	Reverse	5’CGGAGTTTGGGTTTGCTTGTC3’
*CXCL1*	Forward	5’TTTTGAAATGTCAACCCCAAG3’
	Reverse	5’GATCTCATTGGCCATTTGCT3’
*CXCL8*	Forward	5’TCAGAGACAGCAGAGCACAC3’
	Reverse	5’GGCAAAACTGCACCTTCACA3’
*Ifnα*	Forward	5’AAAGAAATGTAAGAAAGCTTTTGATGA3’
	Reverse	5’TACACTTTGGCTCAGGACTCATTT3’
*Ifnβ*	Forward	5’TGGGAGGCTTGAATACTGCCTCAA3’
	Reverse	5’TCCTTGGCCTTCAGGTAATGCAGA3’
*Icam1*	Forward	5’CAATTTCTCATGCCGCACAG3’
	Reverse	5’AGCTGGAAGATCGAAAGTCCG3’
*Vcam1*	Forward	5’TGAACCCAAACAGAGGCAGAGT3’
	Reverse	5’GGTATCCCATCACTTGAGCAGG3’
*E-selectin*	Forward	5’AGCAGAGTTTCACGTTGCAGG3’
	Reverse	5’TGGCGCAGATAAGGCTTCA3’
*Cd31*	Forward	5’CAAACAGAAACCCGTGGAGATG3’
	Reverse	5’ACCGTAATGGCTGTTGGCTTC3’
*H1N1 M*	Forward	5’GGTGTCACTAAGCTATTCAA3’
	Reverse	5’CAAAAGCAGCTTCTGTGGTC3’
*HPRT*	Forward	5’GCAGACTTTGCTTTCCTTGG3’
	Reverse	5’AAGCAGATGGCCACAGAACT3’

### 2.5 Western blotting

Cultured HPMEC were washed with cold PBS containing protease and phosphate inhibitor cocktail tablets (Roche, IN, USA). Then cells were lysed with RIPA buffer containing protease and phosphate inhibitor cocktail (Roche, IN, USA). After a cycle of freeze/thaw, lysates were sonicated and centrifuged to obtain protein supernatant. Protein concentrations were determined by bicinchoninic acid assay using a commercial kit (ThermoFisher, MA, USA). Samples were denatured by heating at 95°C for 5 min after addition of 10% β-mercaptoethanol. Equal amounts of protein were separated by electrophoresis and then transferred on polyvinylidene difluoride membranes. Antibodies with indicated titrations were incubated with membranes blocked with 5% skim milk over night at 4°C. After incubation with horseradish peroxidase (HRP)-conjugated secondary antibodies for 1 h at room temperature, the bands were visualized by using enhancing chemiluminescence (ECL) system (Amersham Biosciences, PA, USA). Densitometry analysis was performed using ImageJ software (http://imagej.nih/gov/ij/)).

### 2.6 shRNA silence and overexpression of target genes

HEK293 cells (1.6×10^4^/well) were added to a 96-well plate in order to screen the silence efficiency of different vectors. The next day of seeding, media was removed, and 110μl of fresh media containing 8 μg/ml Hexadimethrine bromide (Sigma-Aldrich) was added to each well. Lentiviral particles containing target sequences of shRNA were added to wells, and the plate was incubated for 18–20 h in an incubator at 37° C with 5% CO_2_. Lentiviral particles then were removed, and fresh media was added for a culture of 24h. The next day, media was changed with media containing puromycin (Sigma-Aldrich) for another 24-h culture. Media was refreshed with media containing puromycin every 3 days. The knockdown efficiency was checked by Western blotting. Once effective vectors were selected, the same strategy was used in HPMEC. The overexpression of target genes in cultured HPMEC was performed by using GFP-tagged S1PR1 or β-arrestin 2 respectively, with respect to a published protocol [14].

### 2.7 Statistical analysis

Data were presented as mean±SEM (the standard error of the means). The differences between groups were analyzed by student’s *t* tests or ANOVA followed by Bonferroni’s post hoc tests. Statistical analysis was performed by GraphPad Prism 5 Windows Edition (GraphPad Software, CA, USA). A *p* value of less than 0.05 was considered statistically significant.

## 3. Results

### 3.1 H1N1 infects cultured HPMEC cells effectively

**Fig 1:**
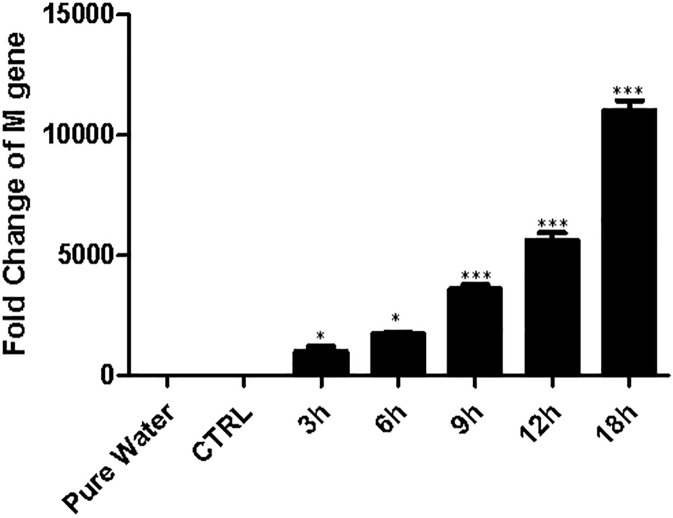
Influenza virus A H1N1 infects cultured HPMEC effectively. Cultured HPMEC were infected with H1N1 or vehicle (CTRL) for 1 hour, and then incubated for different periods. The expression of virus M gene in HPMEC was assessed by qPCR (30 cycles). The expression of M gene increases as culture time prolongs, indicating H1N1 infects cultured HPMEC effectively. *, p<0.05; ***, p<0.001. n = 6, from one of three repeated experiments.

First, we checked the effectiveness of H1N1 infection in cultured HPMEC cells. Cells were infected with H1N1 or the vehicle for 1 h, and then cultured for designated time periods. By the end of culture, cells were collected after thorough washes by cold PBS. Total mRNAs were isolated by using a commercial kit. The mRNA levels of H1N1 M gene were checked by quantitative PCR as an indicator for H1N1 infection. In agreement with a published paper that influenza viruses infect endothelial cells and replicate in these cells [15], the relative fold change of H1N1 M gene increased with a time-dependent manner (Fig 1), suggesting H1N1 viruses infect HPMEC and replicate in cultured HPMEC *in vitro* as culture time prolongs. Infected cells demonstrated good viability after infection (S1 File and S1 Fig). We also infected H1N1 virus to A549 cells following a published paper as a positive control. As shown in S2 Fig, A549 cells were infected with H1N1 effectively, too. H1N1 virus had an increased effectiveness infecting A549 cells compared to HPMEC.

### 3.2 Endothelial cells infected with influenza A H1N1 produced increased levels of pro-inflammatory cytokines, chemokines and cellular adhesion molecules

**Fig 2:**
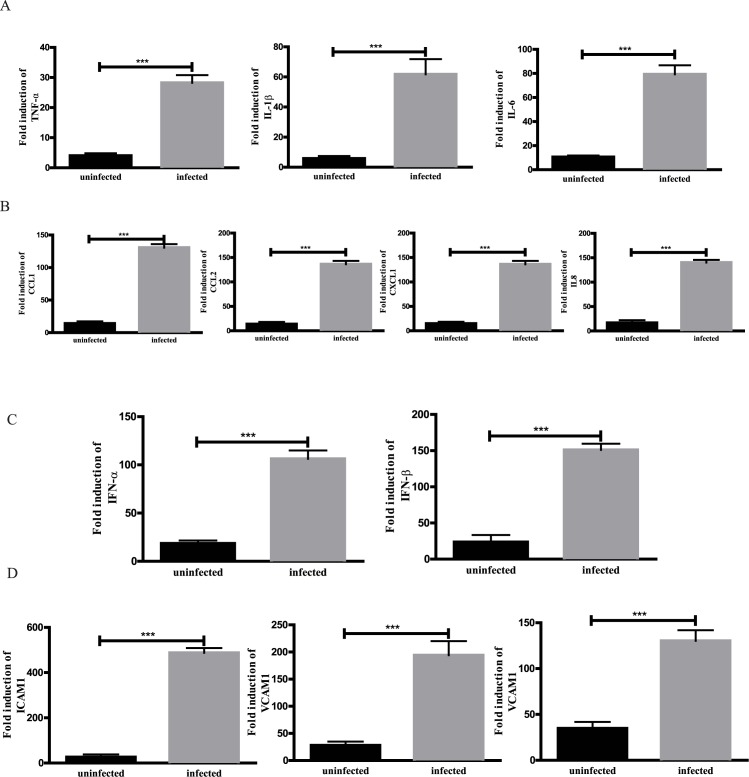
The expression profile of cultured HPMEC infected with H1N1. HPMEC were infected with H1N1 for 1 h, then cultured for 24h. The expression of target mRNAs was measured by qPCR. Uninfected cells were used as controls. A. The expression of profile of cytokines, B. chemokines, C. interferons, and D. adhesion molecules. n = 4. ***, p<0.001.

Endothelial cells are an important component of inflammation, as they control the amount and types of leukocytes recruited in the inflamed sites [4]. However, the effects of influenza A H1N1 in cultured HPMEC remain unknown. As described in Materials and Methods, we infected HPMEC with H1N1. After a period of 24 h of culture after the infection, cultured HPMEC were used for analysis of interested gene mRNA expression. As demonstrated in Fig 2, compared to uninfected cells, virus-infected HPMEC had increased levels of pro-inflammatory cytokines (TNFα, IL1β, and IL6), chemokines (CCL1, CCL2, CXCL1, and IL8), type I IFN (IFNα and IFNβ), and cell adhesion molecules (ICAM1, VCAM1, and E-selectin). The fold changes of cytokines in infected HPMEC ranged between 7 and 70, whereas chemokines had an increase about 100 folds. The most significant alterations were seen in the products of cellular adhesion molecules, indicating the importance of these molecules in inflammation and the interaction between endothelium and leukocytes, which is the essential of inflammation. Notably, mRNA levels of CD31, an endothelial cell marker, did not show a significant change between uninfected and infected cells (data not shown), suggesting viral infection does not affect the phenotypes of endothelial cells. Taken together, these results revealed that endothelial cells act as sentinel cells to detect the presence of virus and initiate the process of inflammation by secreting pro-inflammatory cytokines, chemokines, and IFNs. More importantly, endothelial cells upregulate the expression of cellular adhesion molecules in order to recruit leukocytes in responses to viral infection.

### 3.3 HPMEC express S1PR1 and response to CYM5442

**Fig 3:**
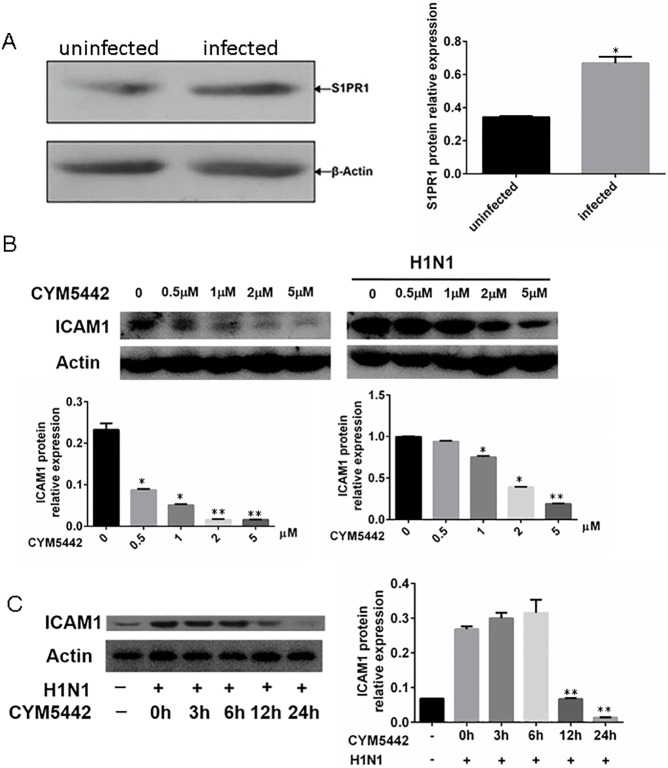
The S1PR1 agonist CYM5442 inhibits elevated ICAM1 expression in HPMEC infected with H1N1. HPMEC infected with H1N1 were treated with CYM5442 with different dosages and time periods. The expression of ICAM1 was measured by Western blotting at the end of stimulation. The representative images of one of tripled experiments were illustrated. A. HPMEC express S1PR1, which is upregulated upon H1N1 infection. B. CYM5442 inhibits the upregulation of ICAM1 in HPMEC infected with H1N1 with a dosage-dependent manner. *, p<0.05; **, p<0.01, compared to cells treated with vehicle. C. At the dosage of 2μM, the suppressive effect of CYM5442 on ICAM1expression in infected HPMEC is time-dependent. **, p<0.01, compared to infected cells treated with vehicle.

In agreement with a previous paper [6], we observed HPMEC expressed S1PR1 at both the mRNA and protein levels, and viral infection increased the expression of S1PR1 (Fig 3A). We then treated HPMEC with the S1PR1 agonist CYM5442. Our preliminary results showed that CYM5442 inhibited the upregulation of mRNAs of pro-inflammatory cytokines, chemokines, IFNs, and cell adhesion molecules with a dosage-dependent fashion (data not shown). Given that CYM5442 had the most significant suppression on ICAM1 in our experimental settings compared to other pro-inflammatory factors, and ICAM1 is essential for endothelial cell biology in terms of controlling neutrophil recruitment [8], we decided to focus on the expression of ICAM1 in cultured HPMEC.

As demonstrated in Fig 3B, CYM5442 treatment decreased the expression of ICAM1 with a dosage-dependent manner. Based on our preliminary results, the optimistic concentration of CYM5442 is 2μM, we also checked the suppressive effects of CYM5442 with a constant dosage at different time points. We showed the representative images of Western Blotting of one of three separate rounds of experiments in Fig 3B, indicating that CYM5442 suppressed the overexpression of ICAM1 in cultured HPMEC infected with H1N1 with a time-dependent manner, and the most significant suppression was seen at the time point 24h. To summarize, CYM5442 treatment inhibits the upregulated expression of ICAM1 in HPMEC infected with H1N1 *in vitro* with a dosage- and time-dependent manner.

### 3.4 Effects of CYM5442 depend on the presence of S1PR1

**Fig 4:**
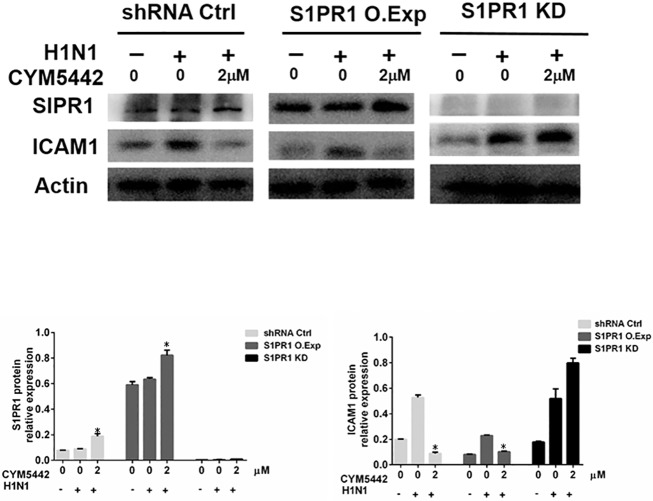
The suppressive effects of CYM5442 on infected HPMEC are S1PR1-dependent. The expression of S1PR1 in HPMEC was knocked in (KI) for overexpression, or knocked down (KD) for loss-of-function assays. HPMEC were infected with H1N1 or vehicle, then treated with CYM5442 or control media. The representative images of Western blotting from one of triplicated experiments are shown. *, p<0.05, compared to infected cells treated with vehicle.

Next, we questioned whether the effects of CYM5442 were S1PR1-dependent. We overexpressed (O.Exp) S1PR1 complementary DNA in HPMEC cells. Meanwhile, HPMEC lacking S1PR1 expression were also created by knocking down (KD) S1PR1 using shRNAs against S1PR1 mRNAs. As demonstrated in Fig 4, HPMEC receiving control shRNA expressed S1PR1, and virus infected cells produced less ICAM1 in responses to CYM5442 comparing to infected cells treated with vehicle. However, CYM5442’s inhibitor effects on ICAM1 expression were enhanced in cells with over-expressed S1PR1, and attenuated in cells with silenced S1PR1 (KD) during H1N1 infection. These data indicate that CYM5442 interacts with S1PR1 to play inhibitory roles upon viral infection, and the effects of CYM5442 depend on the expression of S1PR1.

### 3.5 CYM5442 suppresses NF-κB activation induced by viral infection

**Fig 5:**
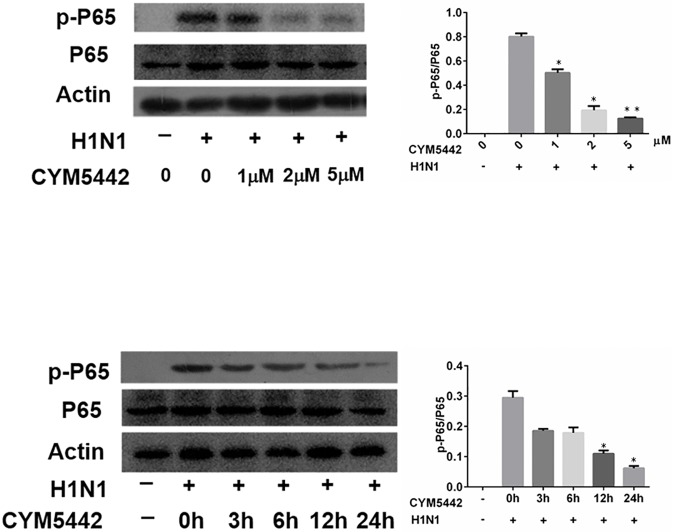
CYM5442 inhibits the activation of NF-κB by a dosage- and time-dependent manner. Representative images of one of triplicated experiments are illustrated. A. Infected HPMEC treated with different dosages of CYM5442 for 24 h. B. Infected HPMEC treated with 2 μM of CYM5442 for different time periods. *, p<0.05; **, p<0.01 compared to infected cells treated with vehicle.

As the NF-κB pathway is a key signaling pathway in endothelial cells activated by inflammatory stimuli, and responsible for the production of ICAM1[16], we assessed the phosphorylation of p65 subunit that is essential for activation and translocation of NF-κB heterodimers. Cells were collected at a designated time point of experiments (24h after viral infection), and whole cell lysates were used for Western blotting assays. We observed that H1N1 infection increased the activation of p65 in cultured HPMEC. However, supplementation of CYM5442 in the culture media decreased the upregulated levels of phosphorylated p65. For a 24-h treatment, as the concentration of CYM5442 increased in the culture media, the suppressive effects on phosphorylated p65 augmented, suggesting CYM5442’s inhibitory effects on phosphorylated p65 in the context of H1N1 infection are dosage-dependent (Fig 5A). The most effective suppression was observed when the CYM5442 concentration was 2μM, which was parallel to that seen in the suppression of ICAM1 expression mentioned above. Again, we stimulated uninfected and infected HPMEC with 2μM of CYM5442 in the culture media for different time periods. Results from Western blotting assay demonstrated that CYM5442 had inhibitory effects on the phosphorylation of p65 in HPMEC upon viral infection with a time-dependent manner (Fig 5B). These data indicate that CYM5442 inhibits the activation of NF-κB in HPMEC infected with H1N1, indicating the mechanisms underlying the suppression of ICAM1 expression.

### 3.6 β-Arrestin 2 is involved CYM5442’s suppression of ICAM1

It remains unknown the mechanisms of CYM5442-induced inhibition of NF-κB activation in HPMEC infected with H1N1. It has been reported that β-arrestins are involved in GPCR signaling pathways with many functions [17]. β-arrestins can also form other GPCR platforms in addition to the canonical heterotrimeric G protein-regulated mechanisms, resulting in activation of intracellular components that suppress NF-κB activation. Then, we hypothesized that β-arrestin might act as the bridge between activation of S1PR1 and inhibition of NF-κB.

**Fig 6:**
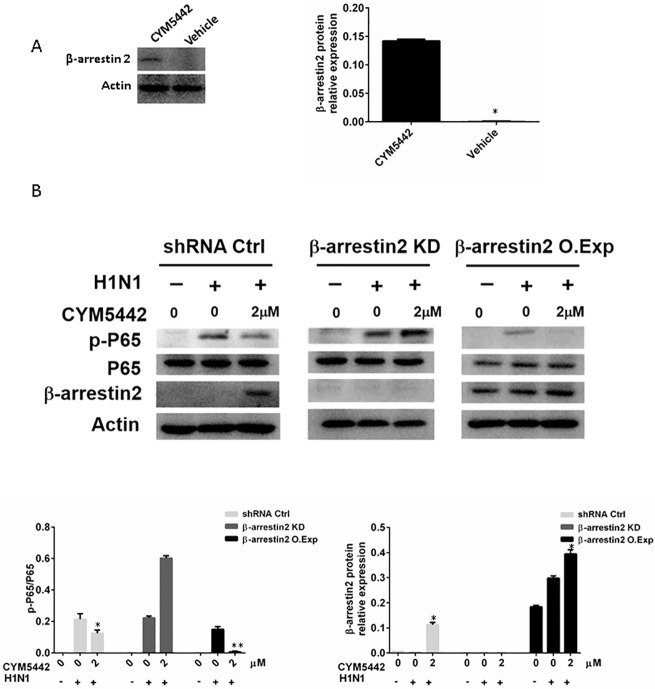
The suppressive effects of CYM5442 on the activation of NF-κB are β-arrestin2-mediated. Representative images of Western blotting from one of triplicated experiments are illustrated. A. CYM5442 increases the expression of are β-arrestin2 in HPMEC infected with H1N1. *, p<0.05. B. Loss of β-arrestin2 blocked the inhibitory roles of CYM5442 on the activation of NF-κB, and overexpression of β-arrestin2 enhanced these suppressive effects. *, p<0.05; *, p<0.01, compared to infected cells treated with vehicle.

Cultured HPMEC were infected with H1N1 or left untreated. Cells were also treated with increased gradient concentrations of CYM5442 or vehicle. Total proteins from cells were used for Western blotting analysis. As shown in Fig 6A, treatment of CYM5442 increased the levels of β-arrestin 2 in HPMEC infected with H1N1, suggesting β-arrestin 2 is involved in CYM5442-induced S1PR1 activation signaling pathway. Then, we utilized shRNA-mediated knockdown (KD) for loss-of-function assays, and overexpression (O.Exp) for gain-of-function of β-arrestin 2, respectively. Virus-infected cells were treated with CYM5442 for 24 h. We demonstrated representative images of Western blotting of one from three independent experiments in Fig 6B. In virus-infected cells, loss of β-arrestin 2 blocked the suppressive roles of CYM5442 on phosphorylation of NF-κB p65, whereas overexpression of β-arrestin 2 enhanced inhibitory effects of CYM5442 on activation of p65 subunit. These data indicate that β-arrestin 2 is involved the CYM5442-S1PR1 signaling pathway and mediates inhibition of NF-κB activation.

## 4. Discussion

**Fig 7:**
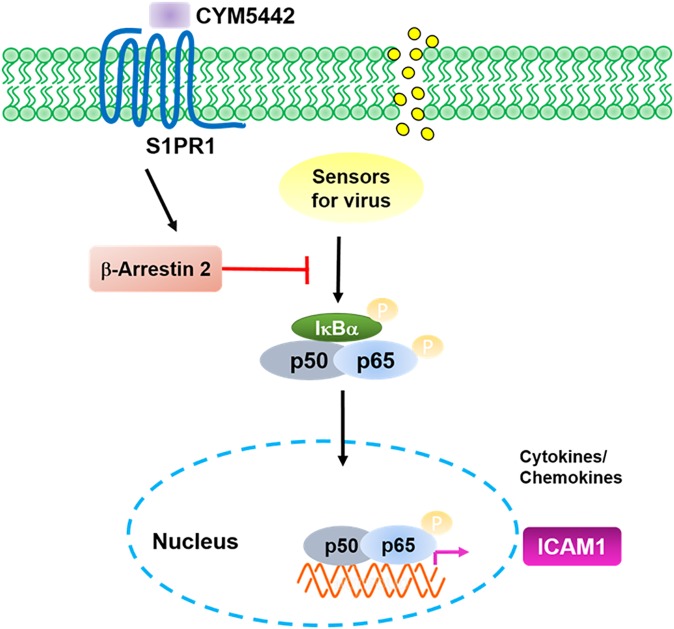
The model of CYM5442-mediated suppression on ICAM1 in infected HPMEC.

In this study, we demonstrated that HPMEC could be infected by H1N1 virus, and responded to viral infection to produce pro-inflammatory mediators including ICAM1. As a major player in the interaction between endothelial cells and leukocytes, ICAM1expression was inhibited by the S1PR1 specific agonist CYM5442 via decreasing the activation of NF-κB. For the molecular mechanism, we found CYM5442 elevated the expression of β-arrestin 2 to inhibit NF-κB activation (Fig 7). Our observations here extended the understanding of regulatory roles of the CYM5442-S1PR1 axis in endothelial cells in the context of inflammation.

Endothelial cells have a central role during inflammation and infection. Activated endothelial cells control the amounts and types of leukocytes infiltrated in inflamed sites [4]. The regulation of endothelial functions is a key step to control the degrees of inflammation. During inflammation, the functions of endothelial cells change, with the characteristics of altered nitric oxide production, increased levels of pro-inflammatory chemokines and cytokines, and an elevation of cellular adhesion molecules including ICAM-1, resulting in the recruitment of immune cells into inflamed sites [8, 18]. Therefore, the upregulation of ICAM-1 in endothelial cells is necessary for leukocyte recruitment.

Sphingolipids, including sphingosine 1-phsphate (S1P), play important roles in many diseases including inflammation, infections, and cancer [10, 11, 19]. S1P functions with specific GPCR called S1PR1-5, and modulates cellular responses [11]. S1PR1 has obtained a great deal of attention, as it shows regulatory functions on endothelial cell activation [6, 20]. Mice with specific deletion of *S1pr1* in endothelial cells had increased expression of ICAM1, whereas mice with upregulated *S1pr1* in endothelial cells showed decreased abundance of ICAM1[20]. In our study, we discovered that S1PR1 in cultured endothelial cells was upregulated after exposure to H1N1 viruses, indicating S1PR1 has some roles during inflammation. Given that addition of CYM5442 in culture medium inhibits products of pro-inflammatory cytokines, chemokines, and ICAM1, the upregulation of S1PR1 is a compensatory effect in order to regulate excessive inflammation. However, exogenous agonists of S1PR1 are needed to regulate inflammation. In agreement with these publications, we demonstrated here the specific S1PR1 agonist CYM5442 decreased the upregulated ICAM1 in endothelial cells upon H1N1 virus infection, indicating the regulatory functions of S1PR1 in endothelial cells also apply in virus infection.

During infection induced by influenza A viruses, endothelial cells in lungs are essential for pathophysiologic responses. The primary cellular target of influenza A virus is epithelial cells in respiratory tracts. Given the intimate distance between epithelial cells and endothelial cells in lungs (an average distance of 0.5μm), it is possible that endothelial cells can be infected by influenza A virus [5]. We infected cultured HPMEC with H1N1 *in vitro*. We checked the mRNA expression of H1N1 M gene in cultured HPMEC after infection showing increased M gene expression as the culture time prolonged, indicating H1N1 infects HPMEC effectively. In a pioneer study, it has been showed that endothelial cells were the major source of pro-inflammatory cytokines and chemokines [6]. Given that activation of S1PR1 inhibited immune cell infiltration in that model [6], it is logical to speculate cellular adhesion molecules expressed by endothelial cells, which are essential for leukocyte recruitment, are also affected by S1PR1 activation. In the current study, we also demonstrated that HPMEC infected H1N1 produced increased levels of ICAM1, which is a key molecule for leukocyte recruitment, suggesting endothelial cells are critical for immune responses induced by H1N1. We also proved infected HPMEC treated with CYM5442 produced decreased levels of ICAM1 compared to infected cells treated with vehicle, suggesting the activation of S1PR1 is an important regulatory pathway for inflammation in lungs induced by viral infection.

Acute respiratory distress syndrome (ARDS) is a fatal complication for patients with influenza virus infection, which leads to multi organ failure and a poor outcome [21, 22]. Endothelial cells, as well as epithelial cells, function as provokers for excessive inflammation in lungs caused by influenza viruses [9]. Influenza virus infection can increase the products of cellular adhesion molecules including E-selectin, P-selectin, and ICAM1, which enhance leukocyte recruitment to alveolus [23, 24]. Infiltrated neutrophils and macrophages damage the epithelial-endothelial barrier via various mechanisms. Influenza virus infection also activates endothelial cells in lungs to produce pro-inflammatory cytokines and chemokines. Infection of H3N2 and H1N1 viruses causes the upregulation of IL-6, CXCL9, and CXCL10 in cultured human umbilical vein endothelial cells [25, 26]. In an animal model of H1N1 virus infection, pulmonary endothelial cells activated by influenza viruses exacerbated disease severity in mice by producing cytokines and chemokines [6]. All these data indicate regulating endothelial functions might be a therapeutic strategy for influenza virus-induced ARDS. Currently, therapies for influenza virus-induced ARDS are non-specific, and most of them aim to relieve symptoms rather than causes [27]. Our findings of regulatory roles of the S1PR1 specific agonist CYM5442 on endothelial cells in influenza virus infection shed a light for new therapeutic routes for influenza virus-induced ARDS, although the application of CYM5442 in clinical practice still needs animal experiments and clinical trials based on the further understanding of physiologic roles of S1PR1.

In conclusion, CYM5442 interacts with S1PR1 to negatively regulate products of pro-inflammatory mediators by decreasing the activation of NF-κB signaling pathways via β-arrestin 2. These results shed a new light for the therapeutic strategies for influenza A virus infection and the subsequent infection-induced complications.

## Supporting information

S1 File Supplementary methods (DOCX)

S1 Fig Virus-infected HPMEC showed similar viability compared to un-infected cells during experimental periods. (TIF)

S2 Fig A549 cells were infected with H1N1 effectively. A549 cells were infected with H1N1 virus as described in Materials and Methods. The mRNA levels of virus M gene were assessed by real-time PCR at designated time points. Experiments were performed triplicated, each of which had 5 wells of cells. *, p<0.05. **, p<0.01. ***, p<0.001.
(TIF)
