# Supplementary material for: Sphingosine 1-phosphate receptor 1 (S1PR1) agonist CYM5442 inhibits expression of intracellular adhesion molecule 1 (ICAM1) in endothelial cells infected with influenza A viruses
Source: PLoS One. 2017 Apr 11;12(4):e0175188. doi: 10.1371/journal.pone.0175188 (PMC5388330; doi:10.1371/journal.pone.0175188)
Supplement: S1 File — (DOCX) [file pone.0175188.s001.docx]

**Supplementary Methods**

Cell Growth Assay

Twenty-four h after infection, infected HPMEC or vehicle-treated cells were collected and seeded in 96-well plates with a density of 1.5$\times$10^4^ cells/well. After 24 h, MTT (5mg/ml in water, 10µl/well), which was purchased from Sigma-Aldrich, was added to each well. Plates then were incubated at 37° C for 4h. Then supernatant was removed and 100 µl DMSO was added to each well. After shaking plates, absorbance values of wells were obtained with a microplate reader at 570nm.
