# Supplementary figures and images for: Sphingosine 1-phosphate receptor 1 (S1PR1) agonist CYM5442 inhibits expression of intracellular adhesion molecule 1 (ICAM1) in endothelial cells infected with influenza A viruses
Source: PLoS One. 2017 Apr 11;12(4):e0175188. doi: 10.1371/journal.pone.0175188 (PMC5388330; doi:10.1371/journal.pone.0175188)

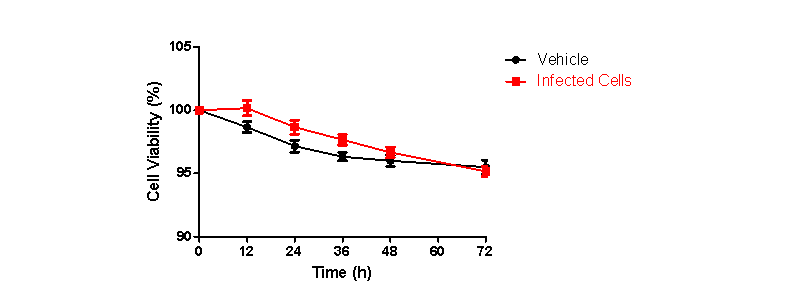

Supplement: S1 Fig — (TIF) [file pone.0175188.s002.tif]

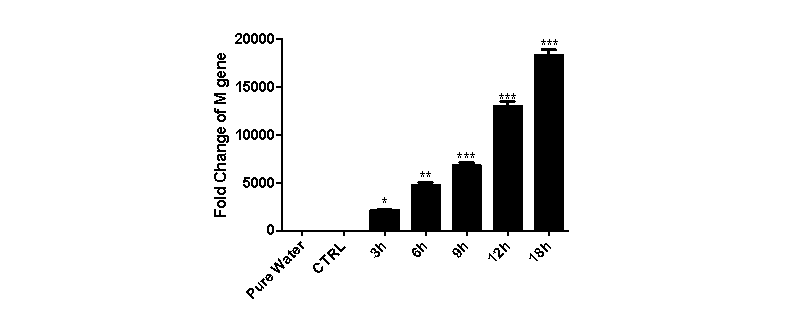

Supplement: S2 Fig — A549 cells were infected with H1N1 virus as described in Materials and Methods. The mRNA levels of virus M gene were assessed by real-time PCR at designated time points. Experiments were performed triplicated, each of which had 5 wells of cells. *, p<0.05. **, p<0.01. ***, p<0.001. (TIF) [file pone.0175188.s003.tif]
